# Impact of Zinc on Oxidative Signaling Pathways in the Development of Pulmonary Vasoconstriction Induced by Hypobaric Hypoxia

**DOI:** 10.3390/ijms23136974

**Published:** 2022-06-23

**Authors:** Karem Arriaza, Constanza Cuevas, Eduardo Pena, Patricia Siques, Julio Brito

**Affiliations:** Institute of Health Studies, Arturo Prat University, Iquique 1100000, Chile; eduardo.enrique.bio@gmail.com (E.P.); psiques@tie.cl (P.S.); jbritor@tie.cl (J.B.)

**Keywords:** zinc, oxidative stress, hypobaric hypoxia, pulmonary vasoconstriction, protein kinase C

## Abstract

Hypobaric hypoxia is a condition that occurs at high altitudes (>2500 m) where the partial pressure of gases, particularly oxygen (PO_2_), decreases. This condition triggers several physiological and molecular responses. One of the principal responses is pulmonary vascular contraction, which seeks to optimize gas exchange under this condition, known as hypoxic pulmonary vasoconstriction (HPV); however, when this physiological response is exacerbated, it contributes to the development of high-altitude pulmonary hypertension (HAPH). Increased levels of zinc (Zn^2+^) and oxidative stress (known as the “ROS hypothesis”) have been demonstrated in the vasoconstriction process. Therefore, the aim of this review is to determine the relationship between molecular pathways associated with altered Zn^2+^ levels and oxidative stress in HPV in hypobaric hypoxic conditions. The results indicate an increased level of Zn^2+^, which is related to increasing mitochondrial ROS (mtROS), alterations in nitric oxide (NO), metallothionein (MT), zinc-regulated, iron-regulated transporter-like protein (ZIP), and nicotinamide adenine dinucleotide phosphate (NADPH) oxidase-induced protein kinase C epsilon (PKCε) activation in the development of HPV. In conclusion, there is an association between elevated Zn^2+^ levels and oxidative stress in HPV under different models of hypoxia, which contribute to understanding the molecular mechanism involved in HPV to prevent the development of HAPH.

## 1. Introduction

Exposure to high altitude (>2500 m) produces a decrease in atmospheric pressure and, in turn, a decrease in the partial pressure of gases, particularly PO_2_, leading to decreased bioavailability of O_2_ in aerobic organisms at the tissue, cellular, and organelle levels; this condition is known as “hypobaric hypoxia” [1,2]. It is important to note that hypobaric hypoxia is distinct from other types of hypoxia, such as normobaric hypoxia, which does not involve a decrease in atmospheric pressure [3]. In addition, exposure to these types of hypoxia leads to activation of a series of physiological mechanisms that compensate for O_2_ deficiency. Under hypobaric hypoxia condition, several pathologies in the cardiovascular system are produced, which will be discussed in this review. It is currently estimated that 81.6 million people worldwide live and work under this particular type of hypoxia, indicating that this is an epidemiological problem worth considering [4].

There are different classifications of hypobaric hypoxia depending on the length of exposure time at a high altitude. The acute hypobaric hypoxia (AHH) exposure model is represented by a short duration of exposure (hours or days), involving primarily mountaineers and tourists. Chronic hypobaric hypoxia (CHH) is another classification and is characterized by permanent exposure to high altitude, for example, among people living at high altitudes (Andeans, Tibetans, and Sherpas, among others) [5,6,7]. However, there is a relatively newly described condition called the Chilean mining model of exposure to high altitude or, in biomedical terms, “exposure to chronic intermittent hypobaric hypoxia” (CIHH), which is based on a shift system (working days at high altitude/rest days at sea level) for a prolonged period (years) [8]. It is important to consider that within the exposure models, there is intra- and interspecies variability in the physiological and pathological responses induced by hypoxia [9,10,11].

The changes induced by AHH can produce mild to chronic pathological alterations (e.g., acute mountain sickness, high-altitude cerebral edema, and high-altitude pulmonary edema), which are rapidly reversible by exposure to normal O_2_ pressures; however, the same does not occur with CHH [12,13], which is characterized by excessive erythrocytosis (chronic mountain disease) and HAPH. Finally, CIHH comprises pathophysiological effects from both AHH and CHH (acute mountain sickness and HAPH) [12].

One of the physiological compensation mechanisms mentioned above that are activated by hypoxia is hypoxic pulmonary vasoconstriction (HPV), where the objective is to redistribute blood to more ventilated areas of the lung [14]; however, in a proportion of individuals, when HPV is exacerbated and permanent over time, processes and molecular mechanisms that lead to vascular remodeling and HAPH, which is characterized by a mean pulmonary artery pressure (mPAP) ≥30 mmHg, become activated [12]. Under CHH conditions, this pathology has a prevalence of 10% [12], and under CIHH conditions, the prevalence is 9% [15]. It is important to note that HAPH generates right ventricular hypertrophy due to pressure overload [16,17], which in some cases can lead to heart failure [18,19].

It has been shown that HPV can be regulated by increases in intracellular zinc concentrations ([Zn^2+^]_i_), which are mediated by S-nitrosylation of the MT protein through NO, inducing contraction of endothelial cells and pulmonary smooth muscle cells (SMC) [20]. In addition, increased [Zn^2+^]_i_ has been shown to be required to modulate the increase in mtROS in hypoxia [21], noting that exacerbated levels of ROS that are not compensated for by antioxidant systems cause increased oxidative stress. Studies have determined that oxidative stress is also related to the processes of pulmonary artery remodeling and subsequent HAPH [22,23].

Therefore, the aim of this review is to determine the possible molecular pathways related to increased intracellular Zn^2+^ (Zn^2+^_i_) levels and oxidative signaling in the development of HPV under hypobaric hypoxic conditions.

## 2. Hypoxic Pulmonary Vasoconstriction

HPV was first described by von Euler and Liljestrand, who postulated that this vasoconstrictor effect corresponds to a physiological mechanism that occurs in response to hypoxia, allowing greater gas exchange [14]. This mechanism has also been described in reptiles and even fish [24,25]. However, studies in rabbits (*Oryctolagus cuniculus*) under acute hypoxic conditions (8% O_2_ × 3 min) have revealed that these animals do not exhibit HPV responses, an interesting topic for future research [26]. Additionally, this vasoconstrictor response could be more exacerbated depending on the type of species; for example, bovines (*Bos taurus*) have a greater vasoconstrictor response than rats (*Rattus norvegicus*) and humans under CHH conditions [27]. With respect to humans, studies have shown that there is variability in HPV responses that are dependent on various factors, such as age, sex, and frequency of exercise, among other antecedents [9,10], which is important to highlight when conducting studies in human populations on this subject.

In particular, studies in the context of AHH (2–10 weeks, over 3800 m) have also reported an increase in mPAP not reaching pathological values [28,29]. However, studies in the context of hypobaric hypoxia, both intermittent (3550 m × 5 d/sea level × 2 d) and chronic (3353 m), have shown that some healthy individuals who are exposed to high altitude respond with exacerbated and permanent HPV processes, developing the pathology of HAPH after remaining at these altitudes for periods longer than 5 months under this condition [30,31]. However, one study conducted by Richalet et al. [8] determined that under CIHH conditions (3800–4600 m × 7 d/sea level × 7 d), there is elevated mPAP in those who develop HAPH due to increased hyperreactivity of the sympathetic system, which decreases to nonpathological values after two years of exposure, although mPAP levels remain elevated compared to those of residents at sea level. Therefore, it can be concluded that HPV develops early (months) in many species, presenting a variety of intraspecies responses. This physiological response involves various mechanisms, which are presented below.

### 2.1. Oxidative Stress

Numerous studies have been conducted under different conditions of normobaric and hypobaric hypoxia, including acute, intermittent, and chronic conditions [18,22,32,33,34,35,36,37,38], where these studies showed that under these conditions, excess production of ROS and reactive nitrogen species (RNS) is generated, altering the redox equilibrium and resulting in the development of oxidative stress [39]. In addition, the severity of oxidative stress could be related to the type of exposure to hypoxia, which is supported by numerous studies reporting that oxidative damage is greater under chronic hypoxic conditions than under acute conditions [32,33,35,36,38,40,41,42].

Particularly, under the condition of chronic hypoxia, studies in animal models such as lambs (*Ovis aries*) at 3600 m and mice (*Mus musculus*) at 10% O_2_ exposed to CHH generate oxidative stress due to increased hydrogen peroxide (H_2_O_2_), superoxide (O_2_^•−^), and hydroxyl radicals (^•^OH). These moieties act on the SMC of pulmonary arteries, regulating tone and vascular remodeling [43,44,45,46]. Particularly, studies involving the pulmonary arteries of rats exposed to CIHH (4600 m × 2 d/sea level 2 d) reveal an increase in biomarkers of oxidative stress (oxidized proteins and lipid peroxidation) and proteins related to oxidative stress, such as NADPH oxidase-2 and NADPH oxidase-4 (Nox2 and Nox4, respectively) in this tissue, contributing to the development of HAPH [22]. Finally, studies in animal models in the context of AHH (8500 m × 3 h) have shown that this type of exposure also generates an increase in biomarkers of damage and oxidative stress (oxidized glutathione and lipid peroxidation), resulting in decreased activity of antioxidant proteins (superoxide dismutase (SOD) and glutathione peroxidase (GSH-PX)) in the lung, which reflects an imminent increase in oxidative stress [47]. Therefore, exposure to hypobaric hypoxia, regardless of type, induced increased ROS and subsequent oxidative stress, contributing to the development of pathologies associated with high altitude [48].

#### 2.1.1. Main Sources of ROS in the Pulmonary Vascular System

In the vascular system, there are various sources of ROS, such as mitochondria (complexes I and III of the electron transport chain), GSH/GSSG and NADH/NAD, and/or the enzymatic complex NADPH oxidase (Nox) [49], where both mitochondria and the NADPH oxidase enzyme complex have been described as the primary source of ROS in the cardiovascular system [50,51]. In addition, studies suggest that both systems contribute to the regulation of HPV independently under normobaric hypoxia [52]. However, to our knowledge, it has not yet been determined which of these two sources is predominant in the generation of oxidative stress between normobaric and hypobaric hypoxia.

Regarding mitochondria as the main source of ROS in this hypoxic condition, numerous studies by Waypa et al. [53,54,55,56] have presented evidence that increased ROS production through the mitochondria is involved in HPV development from acute normobaric hypoxia. In addition, the source of mtROS in the development of HPV under normobaric hypoxia apparently depends on the type of mitochondrial complex analyzed. This has been corroborated by Archer et al. [57], who demonstrated that the use of rotenone, a mitochondrial complex I inhibitor, decreased the production of mtROS, inhibiting HPV under this type of hypoxia (2.5% O_2_ × 6 min). Additionally, the use of antimycin A, an inhibitor of the quinol reductase site of mitochondrial complex III, also inhibited the development of HPV; however, cyanide, which blocks mitochondrial complex IV, failed to abolish HPV. It seems that mitochondrial complexes I and III, in particular, contribute to the development of HPV [57], so it would be important to measure these complexes in HPV under the condition of hypobaric hypoxia.

The NADPH oxidase enzymatic complex presents a varied group of isoforms (Nox1, Nox2, Nox3, Nox4, Nox5, Duox1, and Duox2) that transfer an electron from NADPH to O_2_, generating O_2_^•−^ and/or H_2_O_2_ depending on the isoform [58]. Based on the above, studies have determined that overexpression of the Nox2 and Nox4 isoforms is associated with the development of HPV and pulmonary hypertension under conditions of chronic normobaric hypoxia [59,60,61,62] and hypobaric hypoxia [18].

The first evidence for the role of Nox in HPV came from experiments where iodonium and apocynin compounds (nonspecific inhibitors of NADPH oxidase) were used to attenuate the increase in ROS induced by hypoxia (PO_2_ 8–10 torr, 5% CO_2_, 95% N_2_), the contraction of SMC of pulmonary arteries, and subsequent HPV [63,64]. Marshall et al. [64] provided evidence that the participation of NADPH oxidase in the context of hypobaric hypoxia (5% CO_2_, 95% N_2_) generates increased levels of O_2_^•−^ in the SMC of small pulmonary arteries, inducing HPV. The increase in O_2_^•−^ was inhibited by the administration of diphenyleneiodonium (a specific inhibitor of the enzymatic complex NADPH oxidase). However, the same result was not obtained from the administration of myxothiazol (a mitochondrial inhibitor specific for oxidative phosphorylation). Finally, these studies allow us to postulate that the NADPH oxidase complex is responsible for the production of O_2_^•−^ in the SMC of the pulmonary artery, which could also be involved in the mechanisms of HPV induced by exposure to hypobaric hypoxia.

Activation of the NADPH oxidase enzyme complex has been well described for the Nox2 isoform, where its activation depends on the assembly of its catalytic subunits, corresponding to two membrane subunits (gp91phox and p22 phox), three cytosolic subunits (p47phox, p67phox, p40phox), and the G Rac protein [65,66]. It has been shown that NADPH activation is mediated by protein kinase C (PKC), which phosphorylates the p47phox subunit, allowing the transfer of cytosolic subunits to the membrane, activating the enzymatic complex and increasing the production of ROS [67]. Specifically, the PKCε isoform [20] is a key signaling regulator in the development of HPV [68], which will be discussed later.

There are studies in acute (1% O_2_, 5% CO_2_ × 5 min) and chronic normobaric hypoxia (10% O_2_ × 3 weeks) in which a possible interaction between mitochondrial activity and the activation of NADPH oxidases has been determined, establishing that ROS derived from mitochondria activate the NADPH oxidase enzymatic complex (specifically the Nox2 isoform), generating increased levels of ROS and intracellular calcium concentrations ([Ca^2+^]_i_), triggering the contraction of pulmonary SMC [69]. In addition, Liu et al. [70] previously demonstrated that in conditions of chronic normobaric hypoxia, deletion of the gene encoding the Nox2 protein completely prevents the generation of O_2_^•−^ dependent on NADPH oxidase, preventing pulmonary arterial hypertension induced by chronic hypoxia and vascular remodeling.

Interestingly, some reports in different types of hypoxia (normobaric and hypobaric) and in different study models, such as cell culture and animal models, indicate that the Nox2 enzymatic complex activates pathways through increasing the production of ROS, mediating the development of HPV [61,62,71,72]. In addition, Liu et al. [72] used pulmonary arteries isolated from mice deficient in Nox2 exposed to chronic hypoxia and demonstrated that these mice exhibited significantly reduced levels of O_2_^•−^, which generated decreased HPV. Therefore, based on the findings described above, it is suggested that the source of ROS production derived from Nox2 contributes to greater constriction of the pulmonary artery after exposure to chronic hypoxia.

On the other hand, several studies have indicated that the Nox4 isoform also contributes to oxidative stress by increasing H_2_O_2_ in the pulmonary vascular system under hypoxic conditions [73,74,75]. However, under chronic hypoxia (10% O_2_, 550 mbar), studies have shown some discrepancies with respect to the expression of Nox4 in the development of vasoconstriction of the pulmonary artery and subsequent pulmonary hypertension, indicating that this isoform can mediate different responses depending on the duration of hypoxia and the sex of the animals [76].

However, studies in both patients with other pathologies [77] and mice with pulmonary hypertension induced by exposure to chronic hypoxia have revealed overexpression of the Nox4 isoform [59]. In contrast, Veith et al. [60] determined that mice deficient in Nox4 exposed to acute (1% O_2_ for 180 min) and chronic normobaric hypoxia (10% O_2_ for 21 days) did not display differences in their responses to pulmonary hypertension induced by either model of hypoxia, demonstrating that the generation of ROS from Nox4 does not play a role in the development of HPV or pulmonary hypertension. Therefore, to clarify this controversy, more studies on the activity and expression of this isoform in the context of hypobaric hypoxia are necessary.

Based on all the studies mentioned above, we can hypothesize that the Nox2 isoform has a predominant role in the development of oxidative stress through increased ROS, contributing to the processes of HPV, pulmonary artery remodeling, and pulmonary hypertension. This leads us to different oxidative theories, which are presented below.

#### 2.1.2. Oxidative Theories in the Development of HPV

Two theories on vasoconstriction and the alteration in ROS levels under hypoxic conditions have been proposed, and these theories relate to decreased O_2_ levels and the subsequent impact on mitochondria.

**Figure 1:**
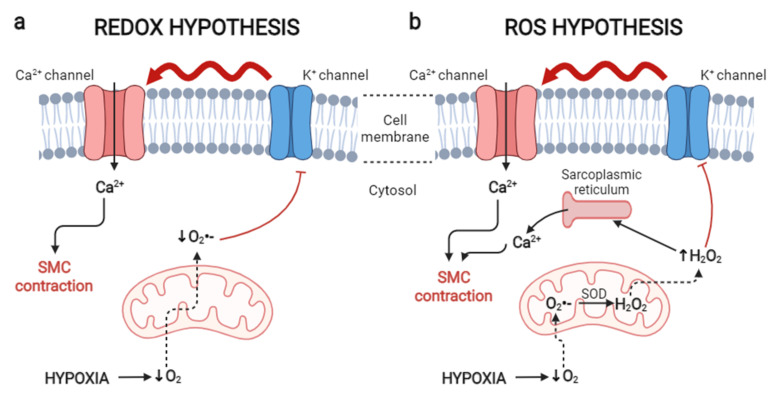
Oxidative theories on hypoxic pulmonary vasoconstriction. (**a**) Theory of smooth muscle cell contraction and subsequent hypoxic pulmonary vasoconstriction induced by a reduced cytosol under hypoxic conditions; (**b**) theory of smooth muscle cell contraction and subsequent hypoxic pulmonary vasoconstriction induced by an oxidized cytosol under hypoxic conditions. SOD: superoxide dismutase; SMC: smooth muscle cell; O_2_: oxygen; O_2_^•-^: superoxide; H_2_O_2_: hydrogen peroxide; Ca^2+^: calcium; K^+^: potassium. Created using BioRender.

The first theory is the “REDOX hypothesis”, where under hypoxic conditions, there is a decrease in the levels of O_2_^•−^ from the mitochondria in the pulmonary artery, decreasing oxidative stress and generating a reduced cytosol. Notably, the reduced state of the cytosol leads to the inhibition of K^+^ channels that are sensitive to PO_2_, SMC depolarization in pulmonary arteries with L-type voltage-dependent calcium (Ca^2+^) channel activation, the triggering of a higher [Ca^2+^]_i_, and the promotion of SMC contraction in the pulmonary artery with subsequent HPV development [49,78] (Figure 1a).

The second theory is known as the “ROS hypothesis” and suggests that under hypoxia, there is increased ROS production from the mitochondria as a result of the electron transport chain, where O_2_, which is reduced to water under physiological conditions, is reduced to O_2_^•−^ under hypoxic conditions, and O_2_^•−^ is then degraded to H_2_O_2_ through the activity of the enzyme superoxide dismutase, resulting in cytosolic oxidative stress, a product of the exacerbated increase in these molecules [79,80]. Consequently, H_2_O_2_ triggers the release of intracellular Ca^2+^ reserves from the sarcoplasmic reticulum and causes inhibition of K^+^ channels, depolarizing the membrane and promoting the entry of extracellular Ca^2+^ through voltage-gated Ca^2+^ channels and inducing increased [Ca^2+^]_i_ that contributes to SMC contraction [81] in the pulmonary arteries, generating subsequent HPV (Figure 1b) [82,83,84].

### 2.2. Bioavailability of Nitric Oxide

Another mechanism that contributes to vasoconstriction is the decreased bioavailability of NO. In the pulmonary vascular system, contraction and dilation of the pulmonary artery is regulated by vasoactive components, such as NO, which is an inherent endogenous vasodilator of the vascular system. The loss of NO bioavailability is related to endothelial dysfunction and vascular pathologies such as pulmonary hypertension [85].

There are different pathways that alter the bioavailability of this endogenous vasodilator and oxidative stress in the development of HPV and pulmonary hypertension. In models of chronic hypoxia, it has been shown that NO reacts with O_2_^•−^ to generate peroxynitrite (ONOO−), which is also considered an oxidative molecule that exacerbates oxidative stress, contributing to vasoconstrictive processes [86,87,88]. Moreover, with respect to NO synthesizing proteins, it has been reported that NO synthases, both inducible (iNOS; inducible nitric oxide synthase) and endothelial (eNOS, endothelial nitric oxide synthase), are capable of generating O_2_^•−^ due to stimulation of the formation of uncoupled eNOS (by a decrease in BH4) [89] or an increase in iNOS activity [90], contributing to vasoconstrictive processes. There are other pathways or molecules related to the processes of vasoconstriction and hypertension, such as asymmetric dimethylarginine (ADMA) [91,92,93], which acts in the body as a competitive inhibitor of NO synthases [94], indirectly contributing to increasing ROS levels through alterations in the typical flow of electrons between the domains of NO synthases, becoming an O_2_^•−^ generator instead of an NO generator in the pulmonary vascular system [95].

Several studies have established alterations in the bioavailability of NO under hypoxia, particularly under CHH (4600 m) and CIHH (4600 m × 2 d/4600 m × 2 d), showing that decreased bioavailability of NO is produced through oxidative stress, which contributes to HPV [23,96]. This is supported in a study by Faiss et al. [37], which showed that exposure of subjects to AHH (3000 m × 24 h) generates decreased NO plasma concentrations and higher oxidative stress in hypobaric hypoxia compared to other types of hypoxia (normobaric hypoxia; 14,7% FiO_2_). However, there are some discrepancies in the alteration of NO levels under hypoxic conditions (1.5% O_2_ × 10 min), as Bernal et al. [20] demonstrated an initial increase in the biosynthesis of NO in the lungs of mice under acute normobaric hypoxia, which causes increased [Zn^2+^]_i_. This increase in turn has been associated with oxidative stress [21], which subsequently decreases the bioavailability of NO.

Therefore, based on the studies discussed above, we can conclude that under hypoxic conditions, there are different molecular signaling pathways (Zn^2+^, ADMA, eNOS, iNOS) that can awaken evident oxidative stress, causing alterations in NO bioavailability and contributing to the development of HPV.

### 2.3. Zinc and Metallothioneins in HPV

Zn^2+^ is considered the second most abundant mineral after iron and is described as an essential mineral in human health that participates as a cofactor for more than 300 enzymes and 2000 transcription factors. Its variety of functions allows it to regulate various physiological processes, and its deregulation can contribute to pathological processes [97,98,99,100].

**Figure 2:**
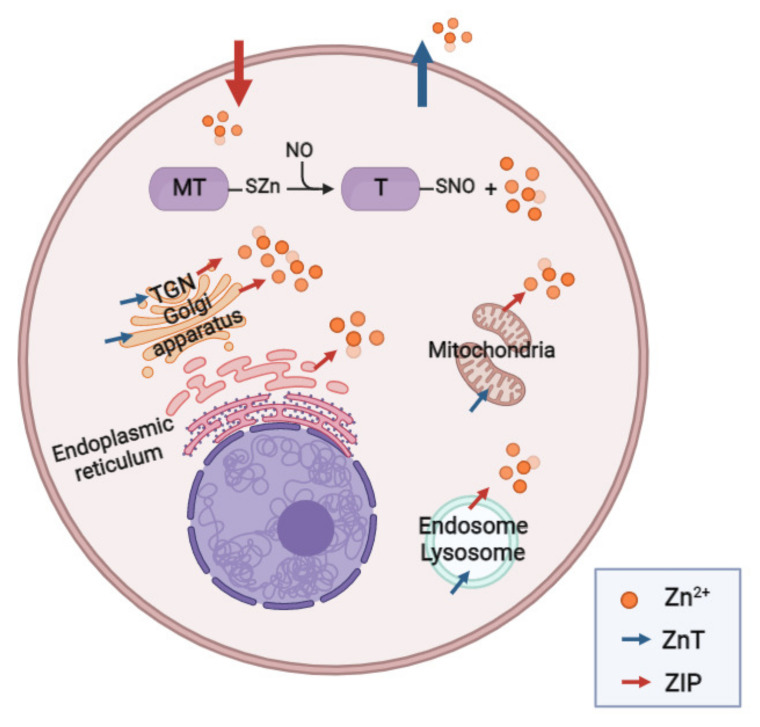
Sources of zinc. TGN: trans-golgi network; Zn^2+^: zinc; MT: metallothionein; T: thionein; NO: nitric oxide; SZn: zinc thiolate; ZnT: Zn transporter; SNO: S-nitrosothiol. Created using BioRender.

In 2008, Bernal et al. [20] proposed a new alternative route of HPV that involves elevated Zn^2+^_i_ levels. In particular, under acute normobaric hypoxia, this type of exposure has been reported to cause increased synthesis of NO, which promotes the posttranslational regulation of the metal-binding protein MT via S-nitrosylation and the addition of an NO moiety to a protein thiol (-SH) of cysteine within the MT, forming S-nitrosothiol (SNO). This results in the release of Zn^2+^ that was previously bound to the cysteine thiol (SZn), and this release results in increased [Zn^2+^]_i_ that contributes to contraction of the pulmonary artery (Figure 2).

This was demonstrated in MT knockout mice exposed to hypoxia, where an 80% reduction in HPV was observed and corroborated with the use of an exogenous vasoconstrictor, such as thromboxane U46619, under normoxic conditions. On the other hand, Zn^2+^ also plays an important role in HPV, as studies in mice exposed to hypoxia with the administration of a specific chelator of Zn^2+^ (N, N, N′, N′-tetrakis (2 -pyridinylmethyl)-1,2-ethanoediamine, TPEN) revealed decreased HPV [20]. Therefore, these studies suggest that both MT and increased levels of Zn^2+^_i_ are the cornerstone of HPV processes.

MTs are low-molecular-weight (6–7 kDa) proteins composed of 20 cysteine residues with two domains (α (residues 32–61) and ß (residues 1–31)). These proteins can bind 7 metal ions divalently, and they have a high binding affinity for Zn^2+^ due to their cysteine residues [101,102,103]. MTs can also be localized to the cytoplasm, nucleus, mitochondria, and endosomes at the extracellular level [104,105,106,107].

MT proteins have 4 isoforms (MT1-4), and their expression is determined by the tissue or cell in which they are located [108,109]. In addition, positive regulation of MT occurs by increases in [Zn^2+^]_i_ and environmental factors, such as hypobaric hypoxia [110], normobaric hypoxia [20], and oxidative stress. One of MT’s functions is antioxidant capacity because the sulfur atoms contained in cysteine residues are capable of eliminating free radicals [111,112], such as H_2_O_2_ [113], O_2_^•−^, and ^•^OH [111], proinflammatory cytokines (such as tumor necrosis factor alpha and interleukin-6) [114] and as part of proliferation, differentiation, and during the immune response (e.g., promoting T-cell survival and proliferation) [115,116,117]. However, despite its antioxidant role, MT is involved in promoting HPV, as mentioned above.

MT transcription is regulated by the metal-sensitive transcription factor (MTF)-1 [118,119,120]. Studies have found that under acute normobaric hypoxia (1% O_2_ × 2–8 h), MT1 expression requires the stabilization of hypoxia-inducible factor-1 alpha (HIF-1α). This was later corroborated by Murphy et al. [102], who determined that in acute hypoxia (1% O_2_; 5% CO_2_), the transcription factors HIF-1α and MTF-1 are required in the promoter zone of the MT1 gene to promote its overexpression, where HIF-1α would play a fundamental role. It is important to note that hypoxia-inducible factor (HIF) is a key transcription factor in the regulation of O_2_ homeostasis. The genes regulated at the transcriptional level by HIF-1α are involved in a variety of processes at the vascular system level, including vascular reactivity, vascular remodeling, glucose metabolism, and cell viability [121,122].

The MT1 and MT2 isoforms are overexpressed in the cardiovascular system under hypoxic conditions (1.5% O_2_, 5% CO_2_, balance N_2_) [20,112], and it has been observed that their overexpression is related to regulatory processes of metabolism, including detoxification of heavy metals and ROS [123,124].

However, in another type of chronic intermittent normobaric hypoxia (20.9% O_2_/8% O_2_ FiO_2_), namely, obstructive sleep apnea (OSA), Zhou et al. [114] found an initial overexpression of MT that occurred approximately 3 days after OSA onset, but the levels subsequently decreased below physiological levels after eight weeks. This can be explained by the occurrence of an imbalance in the antioxidant role of MT, which fails to compensate for the oxidative stress that is generated under prolonged exposure to hypoxia.

Based on the previously mentioned studies above, we suggest that the MT protein is involved in the HPV process by increasing the levels of Zn^2+^_i_. In addition, the MT protein plays an antioxidant role in acute hypoxia models; however, in OSA, this antioxidant protein does not compensate for the exacerbated levels of ROS. Therefore, we hypothesize that the antioxidant activity of this protein depends on the type of hypoxia being studied and that it also plays an important role in the development of HAPH in chronic intermittent hypoxia.

#### 2.3.1. Zinc Transporters

In addition to being regulated by MT, there is a large family of Zn transporters known as ZnT and ZIP that regulate cytosolic levels of this metal. ZIP transporters are also called SLC39A (solute-linked carrier 39) and comprise 14 isoforms (ZIP1-14) that are responsible for increasing Zn^2+^_i_ by promoting its entry from extracellular reserves and, likely, from vesicles into the cytoplasm. In contrast, ZnT transporters, also called SLC30A, comprise 10 isoforms (ZnT1-10) that are responsible for reducing Zn^2+^_i_ through the entry of Zn^2+^ into cytoplasmic vesicles or the extraction of this metal into the extracellular space [125] (Figure 2).

In 2015, Zhao et al. [16] reported the key role of the ZIP transporter, specifically isoform 12 (ZIP12), as a possible therapeutic target to mitigate the development of hypoxia-induced pulmonary hypertension induced by hypobaric hypoxia exposure (4500 m, 12% O_2_). This study demonstrated that this transporter is present in SMC of the pulmonary vascular system and that its expression is dependent on hypoxia. In addition, in CHH-exposed rats with an alteration in the *Slc39a12* gene, which codes for this transporter, decreased Zn^2+^_i_ levels were evident and attenuated the development of pulmonary hypertension.

The findings described above indicate that the involvement of Zn^2+^ and ZIP12 is key in the physiological (HPV) and eventually pathological (HAPH) mechanisms associated with exposure to CHH at the cardiovascular level, opening new avenues for future studies in other models of hypobaric hypoxia.

#### 2.3.2. PKCε and Zinc as Promoters of HPV

The mechanism previously proposed by Bernal et al. [20] proposes that the development of HPV (1.5% O_2_) involves the action of Zn^2+^, NO, MT, and PKCε, where the release of Zn^2+^ from MT has been associated with PKCε activation.

Studies have shown that the activation of PKC in hypoxia is mediated by increased oxidative stress, which was corroborated by a study in which the increase in mtROS under conditions of normobaric hypoxia (air-5% CO_2_ or <1% O_2_) generated the activation of PKCε, which in turn activated the complex. Enzymatic NADPH oxidase phosphorylates and translocates p47*^phox^* to the plasma membrane, further increasing oxidative stress [126,127,128,129]. This was demonstrated experimentally by treatment with the pharmacological inhibitors apocynin (Nox inhibitor) and chelerythrin (PKC inhibitor), along with the use of p47*^phox^* knockout mice, where these inhibitors significantly attenuated the activity of Nox, as well as the increase in the formation of ROS in normobaric hypoxia (1% O_2_, 5% CO_2_, and 94% N_2_). In addition, the genetic deletion of PKCε and inhibition of mtROS with rotenone and myxothiazol (inhibitors of oxidative phosphorylation) treatment blocked the hypoxic effect on the activity of Nox [69]. Therefore, an increase in mtROS levels is essential for the activation of PKCε.

**Figure 3:**
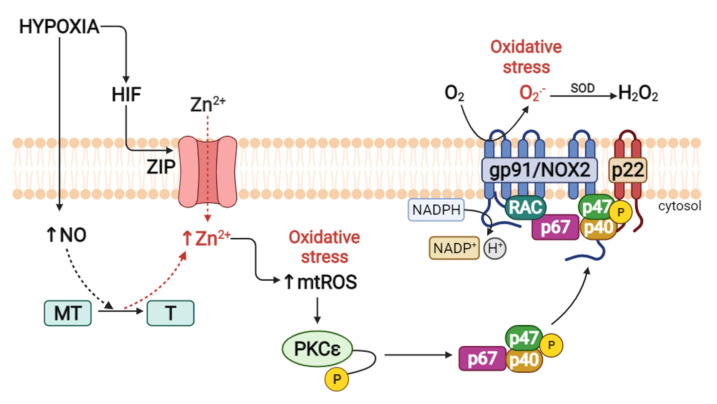
Role of zinc in the activation of NADPH oxidase in hypoxia. NO: nitric oxide; MT: metallothionein; T: thionein; Zn^2+^: zinc; mtROS: mitochondrial reactive oxygen species; PKCε: protein kinase C epsilon; SOD: superoxide dismutase; O_2_^•-^: superoxide; H_2_O_2_: hydrogen peroxide; NADPH/NADP+: nicotinamide adenine dinucleotide phosphate; ZIP: zinc-regulated, iron-regulated transporter-like protein. Created using BioRender.

Regarding mtROS, Slepchenko et al. [21] reported that under conditions of normobaric hypoxia (chemical hypoxia; sodium dithionite (DT) in oxygen- and glucose-deprived (OGD) HEPES buffer), the increase in mtROS levels requires previous accumulation of [Zn^2+^]_i_ since Zn^2+^ is fundamental for the phosphorylation processes of Nox assembly (Figure 3), confirming previous studies. On the other hand, other authors indicate that the activation of Nox mediated by PKC also causes increased intracellular O_2_^•−^ [130] (Figure 3).

In addition, pharmacological and genetic studies in hypoxia (1% O_2_, 5% CO_2_, and 94% N_2_) have shown that PKCε is an important isoform mediating the increase in [Ca^2+^]_i_ and the subsequent contraction of newly isolated mouse SMC, which was demonstrated by the inhibition of mtROS production, which inhibited PKCε activation, generating decreased [Ca^2+^]_i_ and decreased SMC contraction [131,132].

**Figure 4:**
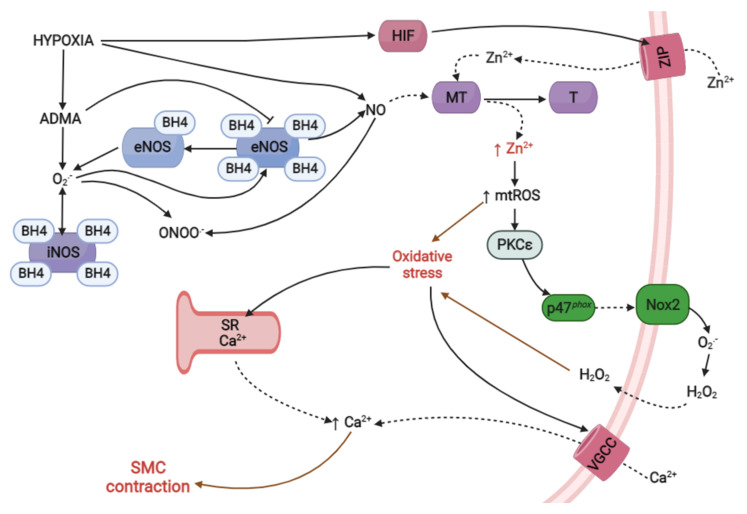
Proposed mechanisms of pulmonary vasoconstriction induced by hypoxia exposure. Zn^2+^: zinc; mtROS: mitochondrial reactive oxygen species; PKCε: protein kinase C epsilon; SMC: smooth muscle cells; HIF: hypoxia inducible factor; ZIP: zinc-regulated; iron-regulated transporter-like protein; MT: metallothionein; T: thionein; O_2_: oxygen; O_2_^•−^: superoxide; H_2_O_2_: hydrogen peroxide; Ca^2+^: calcium; SR: sarcoplasmic reticulum; VGCC: voltage-gated calcium channel; iNOS: inducible nitric oxide synthase; eNOS: endothelial nitric oxide synthase; ADMA: asymmetric dimethylarginine; NO: nitric oxide; ONOO^•−^: peroxynitrite; BH4: tetrahydrobiopterin. Created using BioRender.

These findings allow us to establish that under hypoxic conditions, there is a relationship between increased Zn^2+^_i_ levels, the production of mtROS, and activation of PKCε, contributing to the contraction of SMC in pulmonary arteries through increased intracellular Ca^2+^, as mentioned previously in the ROS hypothesis. This establishes a new molecular pathway that involves the participation of Zn^2+^ and oxidative signaling in the development of HPV, as illustrated in Figure 4.

One of the principal limitations of this review is the limited evidence or studies regarding zinc and oxidative stress pathways and their effects in high altitude exposure; however, it is important to highlight that the lack of studies found supports the statement and pathways presented in this review. Therefore, this research opens new avenues for studying the effects of hypobaric hypoxia. Thus, more studies should be conducted to attenuate the levels of intracellular zinc (e.g., the use of chelators of zinc) and the related molecular pathways (e.g., ZIP12 and PKCε pathways) that are activated under hypobaric hypoxic conditions to mitigate the exacerbated development of HPV triggered in this condition. On the other hand, more studies are necessary that demonstrate the impact of intracellular zinc alterations in hypobaric hypoxia exposure to prevent the development of high-altitude diseases, such as HAPH.

## 3. Conclusions

Exposure to hypoxia generates altered levels of Zn^2+^_i_, the product of the S-nitrosylation of NO on the MT protein, which is related to the development of HPV. Increases in [Zn^2+^]_i_ are also related to oxidative stress, which causes the accumulation of mtROS, activating the p47*^phox^* subunit of the Nox complex through the activation of the PKCε protein and generating exacerbated oxidative stress that ultimately contributes to HPV mediated by Ca^2+^.

Therefore, in future studies it will be important to determine the role that Zn^2+^ plays under different models of hypobaric hypoxia, generating new possibilities to understand HPV processes, along with the identification of possible therapeutic targets such as PKCε, MT, ZIP12, and [Zn^2+^]_i_ in the mitigation of the exacerbated mechanism of HPV, which is one of the mechanisms that induces the development of pathologies such as hypoxia-induced pulmonary hypertension.

## Abbreviations

[Ca^2+^]_i_	Intracellular calcium concentration
[Zn^2+^]_i_	Intracellular zinc concentration
^•^OH	Hydroxyl radicals
ADMA	Asymmetric dimethylarginine
Ca^2+^	Calcium
SMC	Smooth muscle cells
eNOS	Endothelial nitric oxide synthase
AHH	Acute hypobaric hypoxia
CHH	Chronic hypobaric hypoxia
CIHH	Chronic intermittent hypobaric hypoxia
H_2_O_2_	Peroxide
HIF	Hypoxia inducible factor
HIF 1α	Hypoxia inducible transcription factor 1 alpha
HAPH	High altitude pulmonary hypertension
iNOS	Inducible nitric oxide synthase
mPAP	Mean pulmonary artery pressure
MT	Metallothionein
MTF-1	Metal sensitive transcription factor
NADPH	Nicotinamide adenine dinucleotide phosphate
Nox	NADPH oxidase
NO	Nitric oxide
O_2_	Oxygen
O_2_^•^	Superoxide
ONOO	Peroxynitrite
OSA	Obstructive sleep apnea
PO_2_	Partial pressure of oxygen
PKC	Protein kinase C
PKCε	Protein kinase C epsilon
ROS	Reactive oxygen species
HPV	Hypoxic pulmonary vasoconstriction
ZIP	Zinc regulated, iron regulated transporter like protein
Zn^2+^	Zinc
Zn^2+^_i_	Intracellular zinc
